# Brain Measures of Toddlers’ Shape Recognition Predict Language and Cognitive Skills at 6–7 Years

**DOI:** 10.3389/fpsyg.2019.01945

**Published:** 2019-08-23

**Authors:** Kristina Borgström, Janne von Koss Torkildsen, Birgitta Sahlén, Magnus Lindgren

**Affiliations:** ^1^Department of Psychology, Lund University, Lund, Sweden; ^2^Department of Special Needs Education, University of Oslo, Oslo, Norway; ^3^Department of Logopedics, Phoniatrics, and Audiology, Lund University, Lund, Sweden

**Keywords:** N400, shape recognition, language development, ERP, shape bias

## Abstract

While a number of studies have found that an improvement in object shape recognition is associated with language growth in infants and toddlers, no published studies have investigated the longitudinal relation between early shape recognition, and language abilities in later childhood. An electrophysiological measure of semantic processing (the N400) was used to assess shape recognition and general object recognition in a naming context in 20-month-olds. The measures of shape recognition strongly predicted language and cognitive abilities at 6–7 years even after controlling for toddler vocabulary size. The electrophysiological measures of general object recognition were not related to future language or cognitive abilities. These results suggest that early shape recognition abilities may play a role in language acquisition and influence even long-term language outcomes.

## Introduction

The growth of children’s early vocabularies has been systematically linked to the development of the so-called “shape bias”: the tendency to extend newly learned words to objects similar in shape (Landau et al., 1988). While shape bias refers specifically to object categorization in the context of learning novel labels for categories of objects, studies have also shown that the shape bias is related to, and preceded by, a general improvement in object shape recognition (Smith, 2003; Pereira and Smith, 2009; Yee et al., 2012). According to the attentional learning account, word learning, and shape recognition are linked through children’s general ability to detect regularities in their environment (e.g., Colunga and Smith, 2008). Objects tend to be categorized according to shape, and so attending to shape makes it easier to successfully learn new words for objects. Although studies have demonstrated that an improvement in shape recognition, and the development of a shape bias, is associated with an increase in concurrent vocabulary size in toddlers (Smith, 2003; Pereira and Smith, 2009; Yee et al., 2012), so far little is known about the more long-term relevance of early shape recognition for language skills in later childhood. In this longitudinal study, we report how brain and behavioral measures of shape recognition in toddlers relate to language and cognitive ability at 6–7 years.

Important work has demonstrated that supporting the development of a shape bias can have a direct beneficial effect on vocabulary growth. By familiarizing toddlers with novel object categories organized by shape, Smith et al. (2002) were able to accelerate the children’s vocabulary growth outside the lab. One indication that shape recognition may be relevant for more long-term language development is that children with atypical language development (such as late-talking toddlers, and children with specific language impairment or autism) show deficits in shape recognition, and tend not to develop a shape bias (Jones, 2003; Jones and Smith, 2005; Tek et al., 2008; Collisson et al., 2015; Potrzeba et al., 2015). Improved shape recognition and a shape bias normally emerge between 18 and 24 months, or when children reach a productive vocabulary of approximately 50–100 words. In comparison, Collisson et al. (2015) found that 3–4 year-old children at risk for language disorder with larger vocabularies than this did not show a shape bias. Similarly, children with autism seem not to demonstrate a shape bias between ages 2– 6, despite growing vocabularies (Tek et al., 2008; Potrzeba et al., 2015). These results indicate that there are several mechanisms for word learning, and that those involving a shape bias may be more efficient than some of the others.

While the predictive validity of early shape recognition for language outcomes at school entry has not been explored, many studies show that individual differences in early word learning ability predict language skills in later childhood. Vocabulary size in toddlers is often measured using parent report (e.g., Fenson et al., 1994), and such measures significantly predict later vocabulary, grammar, and literacy skills (McGregor et al., 2005; Moyle et al., 2007; Lee, 2011; Rowe et al., 2012). Corroborating evidence comes from real-time measures of word comprehension and word processing efficiency, such as the “looking-while-listening” paradigm which measures the speed of looking to a word’s referent. Studies using this paradigm have shown that vocabulary size in toddlers is related to receptive word processing efficiency, and that speed of word comprehension predicts accelerated productive vocabulary growth and future receptive vocabulary size (Fernald et al., 2006; Fernald and Marchman, 2012). This measure also differentiates “late-talkers” who will move into the normal range of language skills from those who will continue to lag behind (Fernald and Marchman, 2012). Furthermore, toddlers with higher speed of word comprehension were more likely to have higher linguistic and cognitive skills at 8 years, a relationship found to be mediated by working memory skills (Marchman and Fernald, 2008). Given the importance of word learning ability for future language and cognitive development, it seems plausible that a skill such as shape recognition, which is strongly linked to early vocabulary development, would also be relevant to cognitive skills later in childhood.

Electrophysiological methods have also proved useful in investigating how infants and toddlers process and understand the meaning of words in real-time. By presenting words in contexts that are semantically matching or mismatching, children’s understanding of word meanings can be directly measured without requiring a response. Results from such paradigms indicate that individual differences in the N400 effect, an event-related potential (ERP) component which indexes lexical-semantic processing, are associated with language skills. The N400 component is an ERP component elicited by words and other meaningful stimuli, and its’ amplitude is attenuated by any aspect of the context that is associated with, or helps predict, the word. In contrast, cues that are incongruent to the word meaning increase the N400 amplitude (for reviews on the N400, see Lau et al., 2008; Kutas and Federmeier, 2011). The term N400 *effect* (or N400 incongruity effect) is used in this article to refer to the difference between N400 amplitude in incongruous vs. congruous conditions. In infants who have not yet begun to speak, those with larger receptive vocabularies display a larger N400 incongruity effect (i.e., larger difference between incongruous and congruous conditions) (Junge et al., 2012a). Similarly, as toddlers begin to produce words, productive vocabulary size is associated with the N400 effect (Torkildsen et al., 2008; Friedrich and Friederici, 2010; Rämä et al., 2013; Borgström et al., 2015a, b; Cantiani et al., 2017). It has also been found that children at-risk for SLI and dyslexia have reduced or atypical N400 effects as toddlers (Friedrich and Friederici, 2006; Torkildsen et al., 2007; Cantiani et al., 2017). In addition to measuring already established knowledge in children, an ERP picture-word paradigm has been used to simulate novel word learning by pairing pseudowords with novel objects. These studies show that infants and toddlers are able to learn novel word-object associations rapidly, but also that this receptive learning ability is related to differences in productive vocabulary size (Torkildsen et al., 2008, 2009; Borgström et al., 2015b).

Using an ERP picture-word paradigm, we recently reported online brain measures of toddlers’ ability to recognize object shape and object part information during word comprehension (Borgström et al., 2015a). Twenty-month-old children with larger vocabularies demonstrated a larger N400 incongruity effect than children with smaller vocabularies when words were paired with silhouettes of objects, and they were also better able to identify object silhouettes in a behavioral pointing task. In contrast, there was no effect of vocabulary size on the N400 effect to regular pictures of objects which provided details in addition to shape information. Detached parts of objects did not elicit an N400 priming effect at all, regardless of vocabulary size. These results indicate that better recognition of object shape specifically, and more efficient semantic processing of the related words, is associated with larger vocabularies.

In the present study, we assessed the same children at age 6–7 years with a large battery of standardized tests measuring language and other cognitive abilities. This provides the first study of the relevance of shape recognition for language skills in school-aged children, as well as the first study to investigate the longer-term predictive validity of brain measures of online semantic processing in toddlers. In a retrospective study, Friedrich and Friederici (2006) found that toddlers with adequate expressive language skills at 30 months showed presence of an N400 effect to word-picture mismatches already at 19 months. However, children with poor expressive language skills at 30 months did not display the N400 effect when tested at 19 months. Thus, at this age the N400 component appears to be able to distinguish children who will go on to have a typical language development from those who will have language delays. We therefore explored how 20-month-old’s semantic processing during both regular object and shape recognition predicted their language and cognitive skills at 6–7 years. We had two main hypotheses: (1) that toddlers who demonstrate a larger N400 effect in shape conditions (an electrophysiological measure of efficient shape recognition) would have stronger language skills at 6–7 years, and (2) that the relation between shape recognition and school age language cannot be explained by a correlation between school age and toddler language skills or by a correlation between school age language skills and the regular N400 effect (i.e., N400 effect in conditions with regular pictures of objects).^1^

## Materials and Methods

### Participants

The sample size included in the various analyses differed due to many children contributing only to parts of the data set. The ERP data at 20 months suffered the highest attrition rates, and thus the main longitudinal analyses between ERP data at 20 months and language and cognitive tests at 6–7 years were performed on a sample of 23 children.

#### Toddlers

The full toddler sample consisted of 77 children (36 boys) at 20 months of age (±3 weeks). Inclusion criteria were: typical development according to parent report, monolingual Swedish speaking, and full term birth (>36 gestational weeks). Reliable electrophysiological data was obtained from 38 children (17 boys). The other 39 children were excluded from EEG analysis due to fussiness, technical problems, or too few artifact-free trials in one or more of the analyzed conditions. Developmental data from questionnaires was obtained for 74 children. The children returned at 24 months (±3 weeks) for the same experimental procedure with novel stimuli. The present study only reports ERP data from the 20 months session, since this time point best captured the period of emergence of the shape recognition/shape bias skills (50–100 words vocabulary, and in between 18 and 24 months). In addition to the ERP data, the current analyses included measures of vocabulary size from both 20 and 24 months. Participants were recruited mainly through child healthcare centers in and around Lund, Sweden, and an information campaign sent by mail to all children in certain areas close to Lund that would fall within the appropriate age range during the study period. The project was granted ethical approval by the Regional Ethical Review Board, according to the decision DNR-2009-383, and the parents of all participants provided written informed consent.

#### 6–7 Years

Families that participated at 20 months were invited to participate in the follow-up study, and 36 (16 girls) of those contacted agreed. At the time of testing, the children were between 6:03 and 7:09 years old. Twenty-three of these participants had contributed reliable ERP data at 20 months. The parents of all subjects gave written informed consent in accordance with the Declaration of Helsinki. The project was approved by the Institutional Review Board at the Section of Logopedics, Phoniatrics, and Audiology at Lund University.

### Materials

#### Toddler Experiment

The event-related potential experiment at 20 months contained 30 common count nouns and 30 pseudowords which were phonotactically legal in Swedish. Words were recorded in an anechoic chamber by a female voice, speaking in an infant-directed manner, and presented through speakers placed in front of the participants. The visual stimuli consisted of cartoon images^2^ of the objects corresponding to the chosen nouns, and fantasy objects and creatures to be paired with the pseudowords. Two modified versions of the pictures were created, one displaying only a few isolated parts (used in the part condition), and one displaying a black, filled silhouette of the object (used in the shape condition). EEG was recorded with infant versions of the 128 channel hydrocel geodesic sensor nets (Electrical Geodesics, Inc.) connected to a Net Amps 300, with a sampling rate of 250 samples/second, referenced to the vertex. Impedances were kept below 50 kΩ, according to recommendations from the manufacturer. In addition to the experimental data, parents reported on their children’s receptive, and productive vocabulary size using the Swedish version of the MacArthur-Bates Communicative Development Inventories (SECDI) (the infant version “words and gestures” which includes measures of both receptive and productive vocabulary, and the toddler version “words and sentences” which only contains measures of productive vocabulary). The toddler version “words and sentences” was once again completed when the children were 24 months (±3 weeks), but not “words and gestures” since this version does not provide norms for 24-month-olds). Thus, three language measures from the toddler period were available: receptive vocabulary at 20 months, and productive vocabulary at 20 and 24 months.

#### 6–7 Years

Tests were chosen from several different standardized batteries, to measure the following linguistic and cognitive abilities: receptive and expressive language abilities, verbal short-term and working memory, communicative skills (by parent questionnaire), and fluid intelligence. See Table 1 for a list of the chosen measures. The majority of measures were selected from different standardized cognitive and linguistic tests for children. The tests are commonly used and are valid and reliable assessment tools for Swedish-speaking children according to clinicians in both speech pathology and psychology. The tests were also considered relevant for the particular lexical focus of this study. Reading tests were not included. However, we included tasks considered to predict or correlate with reading skills, such as tasks tapping implicit (non-word repetition and speeded naming) and explicit (segment subtraction) phonological processing. This was motivated by the fact that some participants were still in pre-school or in preparatory class at the time of testing. Variation in early reading skills instruction (before grade one) in Sweden is still considerable.

**TABLE 1:** Test battery at 6–7 years.

**Measure**	**Source**
Sentence comprehension	Test for reception of grammar (TROG-2) (Bishop, 2009)
Expressive vocabulary	Clinical evaluation of language fundamentals (CELF-4) (Semel et al., 2003)
Similarities (expressive)	CELF-4
Similarities (receptive)	CELF-4
Digit span forward	CELF-4
Digit span backward	CELF-4
Speeded naming	A developmental neuropsychological assessment (NEPSY-II) (Korkman et al., 2007)
Word fluency	NEPSY-II
Non-word repetition	Sound information processing system (SIPS) (Wass et al., 2005)
Segment subtraction	Illinois test of psycholinguistic abilities (ITPA-3) (Hammill et al., 2001)
Auditory analysis	ITPA-3
Overall communication skills (parent report)	Children’s communication checklist (CCC-2) (Bishop, 2003)
Fluid intelligence	Raven’s colored progressive matrices (CPM) (Raven et al., 1990)

### Procedure

#### Toddler Experiment

Children sat on their parent’s lap, with a screen placed around them in order to block out distractions. Pictures were presented on a 17-inch computer screen (34 cm × 27 cm) approximately 35 cm from the child, and words were presented from a speaker next to the screen. Breaks were taken between blocks if necessary, with the possibility of showing a short video clip to recapture the child’s attention. A camera placed in front of the child recorded the child’s behavior throughout the experiment, allowing for exclusion of trials where the child was inattentive.

**FIGURE 1:**
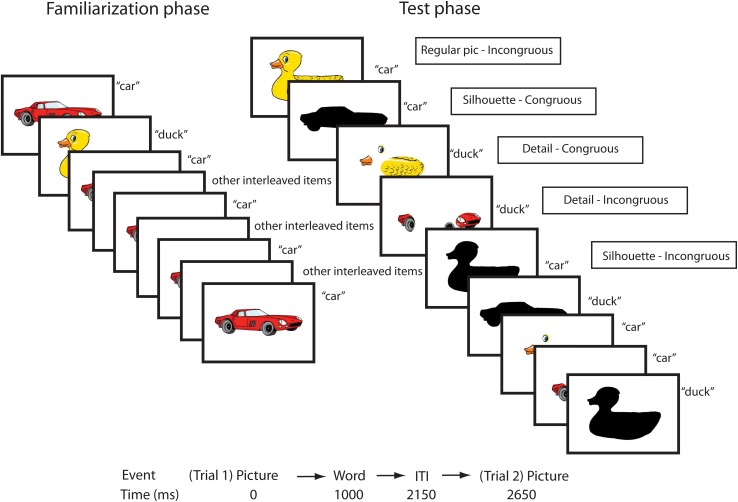
Overview of experimental design containing real words and objects. Figure reproduced from Borgström et al. (2015a).

The stimuli were organized into ten independent blocks, with each block containing three real words and objects and three pseudowords and novel objects. Please see Figure 1 for an illustration of the experimental design. Note that, although the experiment included both real words and their referents, and pseudowords paired with fantasy objects, this paper only reports data from the real word conditions as predictors for future cognitive skills. Each picture-word pair was presented five times in a pseudo-randomized order. The first trial in each block was always a real word, there was at least one interleaved item in between item repetitions, and at most two successive real word trials or pseudoword trials. Each block ended with a test phase, where the picture word pairings were switched. Each word/pseudoword was now presented together with one of the other pictures from the same block, yielding an incongruous pairing. Real objects were always paired with other real words, and novel objects with other pseudowords. Within each block, all words and pseudowords began with different syllables, so that each auditory stimulus could be differentiated from the other words or pseudowords at word onset. In addition to the incongruous pairings, the test phase also included conditions where the modified versions of the original pictures were presented with congruous and incongruous words (modified versions of both real and novel objects were presented). Thus, the test phase contained 10 conditions: regular real objects incongruous, regular novel objects incongruous, real object shapes congruous/incongruous, novel object shapes congruous/incongruous, real object parts congruous/incongruous, and novel object parts congruous/incongruous. The number of trials per condition were 30. Only four real object conditions (regular and shape congruous/incongruous) were analyzed in the present study. In the original study, a condition displaying object parts (such as the eye, beak, and wing of a duck) was included in order to test whether children who had not yet developed a shape bias more easily recognized the objects from isolated parts/details than shape. However, since the results showed that the children overall had great difficulties recognizing these pictures as objects, and that performance in this condition was uncorrelated with vocabulary, this condition was not included in the present longitudinal study. Participants were presented with one of two different trial lists, containing the same stimuli but in different pairings (for the pseudowords and novel objects), and presentation order. Pictures were presented for 2150 ms, with a word onset of 1000 ms after each picture onset, and an inter-trial interval of 500 ms showing a white screen.

#### Follow-Up Test at 6–7 Years

The test battery was administered in the children’s homes. It took between 2 and 3 h to complete, including breaks. Sometimes a caregiver was present during testing, but instructed to remain silent. The tests were administered in the following order: TROG-2, word fluency, expressive vocabulary, segment subtraction, Raven’s colored progressive matrices (CPM), non-word repetition, digit span forward and backward, similarities receptive and expressive, speeded naming, and auditory analogies.

### Analysis

#### Toddler ERP Data

EEG data was pre-processed according to description in Borgström et al. (2015a). Results from the traditional ERP analyses reported in Borgström et al. (2015a) revealed a long-lasting and broadly distributed incongruity effect in the shape conditions. Importantly, different time windows of the shape incongruity effect, in different spatial locations, were found to correlate with different behavioral measures. This raised the hypothesis that the large effect in fact was the result of more than one component. In order to explore this hypothesis, and potentially obtain purer ERP measures as predictors of cognitive skills, we re-analyzed the entire 20-month ERP data set using a principal components analysis (PCA) approach. Prior to the PCA, we also explored the longitudinal relations between the original ERP measures (mean amplitude in a certain time window) and key follow-up measures. We found that the shape incongruity effect at parietal sites (between 500 and 900 ms) correlated with several of the follow-up measures, both language and other cognitive tests. The shape incongruity effect across central sites, in the same time window, correlated only with tests of working memory specifically. Meanwhile, the measure that in the original study correlated with concurrent productive vocabulary (shape incongruity effect at central sites between 700 and 900 ms) was uncorrelated with future vocabulary. A table overview of the longitudinal correlations with the original ERP measures can be found in Supplementary Material 1. Overall, we found the longitudinal results using the original ERP measures to be difficult to interpret, and therefore decided to re-analyze the data using PCA. As Dien and Frishkoff (2005), (p. 189) argue, PCA has the potential of simplifying complex data and the factors obtained “are considered more likely to represent pure signal (i.e., brain activity) as opposed to noise.” The PCA was performed on the original full sample (*n* = 38) of 20-month-olds that contributed at least 10 artifact-free trials per condition. As is recommended by Dien (2012), individual averages including all conditions (shape and regular, congruous, and incongruous), all channels, and the full epoch including the baseline period, were entered into the analysis, which was performed using the ERP PCA Toolkit for Matlab.

Also following recommendations by Dien (2012), we performed a temporal PCA with a Promax rotation, with a covariance relationship matrix and Kaiser weighting. This analysis resulted in two main temporal components (see section “Results” for details), which were used in further analyses, one corresponding to a typical N400 component, and one later component. The measure used for statistical analyses was the mean amplitude of a component across the entire epoch (−100 to 1250 ms) time-locked to the onset of the word stimulus. A region of interest was selected for each effect, based on visual inspection of the largest differentiation between congruous and incongruous conditions. For the measures of the shape and regular N400 effects, the entire parietal region was selected (the same electrodes as in previous publications on this data set, Borgström et al., 2015a, b, 2016). The shape late negativity effect was calculated based on the entire central region (also corresponding to the electrode groupings in previous publications). The regular late positivity effect, however, had a parieto-occipital distribution, and therefore a new region of interest covering the mid parieto-occipital area was created (electrodes 62, 67, 71, 72, 76, and 77). The mean amplitude of all electrodes in each region of interest was calculated for use in further statistical analyses. Repeated measures ANOVAs were performed to confirm statistically significant effects of congruity (1 factor: Congruity, 2 levels). In order to analyze individual differences in congruity effects, we calculated difference scores for each effect, always subtracting the congruous condition from the incongruous condition.

#### Language and Cognitive Skills at 6–7 Years

Results were scored according to each test’s manual, and normed when appropriate test-norms were available. The results section reports scores expressed in percentiles. Speeded Naming and non-word Repetition did not have available norms, so results from these tests are reported as raw scores. The Speeded Naming test produces two measures: accuracy and speed.

The age-normed results from the Raven’s CPM test showed a strong ceiling effect, with a mean score of percentile 83, and 16 participants obtaining the top score of percentile 95. The norms for this test are old (from 1982) and known to be too lenient. In order to obtain an age-adjusted measure of the Raven’s score that better differentiated between participants, we transformed the raw scores by performing a linear regression with age as an independent variable. The residuals were then saved as a new variable, with an added constant of 5 to remove negative numbers. By this procedure, the effect of age could be removed from the raw scores, creating a new variable that was normally distributed.

#### Longitudinal Analyses

Statistical analyses were performed using IBM SPSS Statistics, version 22. All measures were checked for normality of distribution using the Shapiro-Wilks test. Only two school-age measures were normally distributed (Raven’s CPM raw scores, and non-word repetition), while all toddler measures except Productive Vocabulary at 20 months were normally distributed. To adjust the positively skewed distribution of this variable, a log10 transformation was applied. To explore the longitudinal relations between all toddler and school age measures, we first calculated bivariate correlations using Spearman’s rank order correlation for non-normally distributed variables, and Pearson’s correlations for normally distributed variables. A correlation matrix for all individual variables can be seen in Supplementary Material 2.

In order to reduce the number of variables at 6–7 years, and also facilitate interpretation of the longitudinal relations, further analyses focused on the eight variables that correlated with the toddler ERP measures (see Table 2 in section “Results”). One of these variables was Raven’s CPM. This was the only test that did not directly measure language skills or working memory, but was rather selected as a measure of fluid intelligence. The other seven measures that correlated with the ERP measures were related to different aspects of language processing and verbal working memory, and also correlated with each other. These seven measures were included in a PCA with the purpose of reducing the variables to fewer factors. The method used was a PCA with a promax rotation resulting in two factors with an eigenvalue above 1. A promax rotation was chosen because this allows non-orthogonality of the factors. Since the cognitive constructs underlying the factors should be assumed to correlate, we found it reasonable to allow the factors to correlate as well.

**TABLE 2:** Descriptive statistics for all behavioral measures.

**Measure**	**Type**	***n***	**Median**	**Mean (SD)**	**Range**
**Toddler**					
20 m rec. vocabulary	Raw	33	183	190.88 (69.15)	86–319
20 m prod. vocabulary	Raw	33	60	108 (102.25)	7–391
24 m prod. vocabulary	Raw	31	313	290 (161.22)	15–565
**6–7 years**					
TROG-2	Perc	36	70	64.11 (25.92)	1–96
Exp. vocabulary	Perc	36	84	66.44 (29.62)	1–98
Similarities (exp)	Perc	36	37	46.89 (26.38)	5–91
Similarities (rec)	Perc	36	37	41.28 (25.39)	5–84
Digit span forward	Perc	36	25	38.31 (26.10)	2–91
Digit span backward	Perc	36	63	53.94 (23.76)	16–98
Segment subtraction	Perc	36	87.50	67.39 (33.42)	9–99
Auditory analysis	Perc	36	95	78.89 (26.66)	16–99
Word fluency	Perc	36	75	65.94 (27.99)	2–99
CCC2	Perc	36	53.50	54.31 (28.21)	2–97
Raven’s CPM	Perc	36	90	83.19 (19.09)	10–95
Raven’s CPM	Raw	36	26	25.81 (5.47)	13–34
Raven’s CPM raw age-adj.	Residuals	36	5.07	5.00 (0.98)	2.88–6.90
Speeded naming (acc.)	Raw	36	84	102.06 (28.06)	53–135
Speeded naming (time)	Raw	36	150	167 (55.26)	104–305
Non-word repetition	Raw	35	13	13.20 (4.02)	4–22

*Measures are presented in percentile scores when suitable norms were available. Otherwise in raw scores. For Raven’s CPM three different scores are reported for comparison (see section “Materials and Methods” for this methodological consideration).*

The resulting two factors were subsequently used as outcome variables instead of the seven measures that entered the analysis. Further longitudinal analyses focused on how the toddler measures were able to predict these two factors as well as Raven’s CPM as a measure of fluid intelligence. The three outcome variables were tested for normality of distribution using Shapiro-Wilks normality test. While two of the variables were clearly normally distributed, factor 1 fell just below the threshold of statistical significance (*p* = 0.047). However, skewness and kurtosis values were relatively normal (between −0.45 and −0.65), indicating that the factor was reasonably normally distributed. Therefore, parametric statistics (bivariate Pearson’s correlations and multiple regressions) were used in subsequent analyses. Still, comparison analyses were performed entering a transformed version of factor 1 (reflected square root), that yielded results equivalent to analyses involving the original variable.

## Results

### PCA Analysis of ERP Data

**FIGURE 2:**
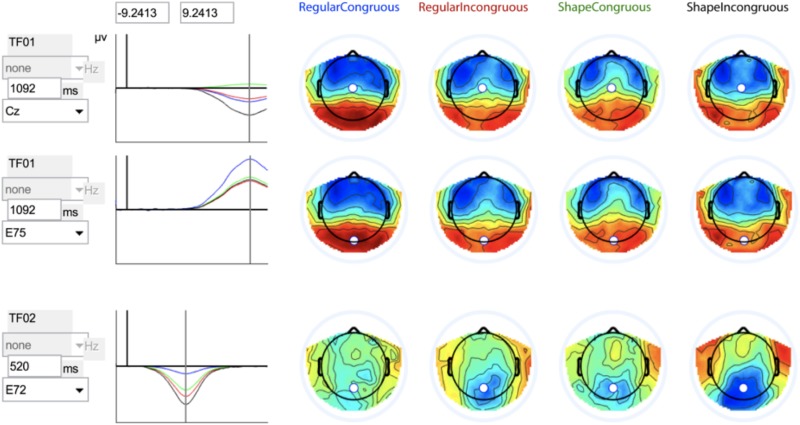
Illustration of the two temporal factors (TF01 and TF02) from the PCA analysis. The figure includes topographical plots of the components in each of the four conditions (regular object vs. shape, and congruous vs. incongruous presentations), where the color blue represents a negative amplitude, and red represents a positive amplitude. The graphs show the temporal properties of the components in each of the four conditions (negative amplitude plotted downward). Electrodes showing the largest effect of incongruity were chosen to illustrate the component waveforms, and for TF01 the topography of this effect differed in the regular object and shape conditions, which is why we display both a central electrode (Cz) for the shape incongruity effect, and parietal/occipital electrode (E75) for the regular incongruity effect.

The temporal PCA produced a total of 16 factors, of which only the first two factors accounted for a variance greater than 0.1 (all others had a variance <0.065). Therefore, only the first two components were further analyzed. Factor one (variance 0.37) was a late component, with a peak latency of 1092 ms. This component had its largest negative peak at channel 14 (frontopolar), and largest positive peak at channel 90 (right occipital). Factor two (variance 0.21) was a mid-latency component with a peak latency of 520 ms, a peak negativity at channel 74 (mid occipital), and a peak positivity at channel 122 (right frontal, F8). These two components were further examined in terms of the experimental effect of congruity (amplitude difference between condition where the word is presented with the correct vs. incorrect object referent), in both the regular object conditions and the shape object conditions. An incongruity effect in the regular object conditions indicates that the child recognizes an object from a typical illustration and knows the correct label for it, while an incongruity effect in the shape conditions is a measure of the child’s ability to also recognize the object from a sparse silhouette representation, i.e., object shape recognition. The mid-latency component (factor 2) showed a congruity effect over parietal and occipital areas, in both the regular and shape object conditions (see Figure 2). Based on the congruity effect, topography and latency, we identified this component as an N400 component. The late component was composed of a negative frontal wave and a positive posterior wave. The effect of congruity (i.e., difference between the conditions) was largest over central electrodes in the shape conditions, with a more negative wave in response to incongruity. Meanwhile, in the regular object conditions occipital electrodes differentiated most between congruous and incongruous presentations, showing a larger positivity in response to congruous presentations. In other words, for shape conditions there was a late central negative effect of incongruity, while for regular objects, there was a late posterior positive effect of congruity. In total, the temporal PCA yielded four experimental effects, since each condition (shape and regular objects) showed an effect in the two temporal factors (Factor 1:N400 and Factor 2: the late negativity/positivity). Below, these experimental effects will be referred to as: shape N400, regular N400, shape late negativity, and regular late positivity.

Repeated measures ANOVAs confirmed that all these congruity effects were statistically significant (see section “Materials and Methods” for details): shape N400, *F*(1,37) = 5.75, *p* = 0.022, *η^2^* = 0.034; shape late negativity, *F*(1,37) = 14.03, *p* = 0.001, *η^2^* = 0.275; regular late positivity*, F*(1, 37) = 4.95, *p* = 0.032, *η^2^* = 0.118. For the Regular N400 effect, the effect across the entire parietal region was not statistically significant, *F*(1,27) = 3.83, *p* = 0.058, *η^2^* = 0.094. Visual inspection showed that in the regular objects conditions, the N400 effect was more localized to the right parietal region, and when including only this region in the ANOVA, the effect was significant, *F*(1,27) = 6.08, *p* = 0.018, *η^2^* = 0.141.

### Cognitive Measures

Descriptive statistics for the cognitive measures at age 6–7 years, as well as 20 to 24 months, are presented in Table 2. For instruments with available published norms, data are presented in terms of percentile scores, and otherwise in raw scores.

### Longitudinal Relations Between Toddler Measures and School-Age Measures

Of the four ERP measures at 20 months, analyses showed that only the two components from the shape conditions correlated with future cognitive measures (see Supplementary Material 1). Parent report of early receptive and productive vocabulary also correlated significantly with several future measures, where productive vocabulary at 24 months in general was a better predictor than receptive vocabulary at 20 months, which in turn was a better predictor than productive vocabulary at 20 months. Two measures from the 6–7-year test battery were uncorrelated with all early measures: CCC-2, and similarities receptive.

The principal components analysis analysis of the selected outcome measures (see section “Materials and Methods”) resulted in two factors. Factor 1 loaded most heavily on the variables Expressive vocabulary and TROG, while the Factor 2 loaded most heavily on the backward digit span test, but also on the forward digit span and speeded naming accuracy measure. From the pattern of loadings, we found it reasonable to conceptualize Factor 1 as “language ability” (receptive and expressive), and Factor 2 as “verbal executive function” (see Table 3 for the PCA pattern matrix). In the discussion we expand our reasoning behind this conceptualization, in particular the concept of executive function. These two factors were reasonably normally distributed (see section “Materials and Methods”), and therefore parametric statistics were suitable in the analyses that follow.

**TABLE 3:** Pattern matrix for the PCA analysis.

**Measure**	**Component 1**	**Component 2**
		
	**Language ability**	**Verbal executive function**
TROG	0.975	–0.154
Expressive vocabulary	0.955	–0.148
Non-word repetition	0.744	0.195
Digit span forward	0.367	0.609
Digit span backward	–0.260	0.921
Similarities expressive	0.540	0.339
Speeded naming (accuracy)	0.072	0.452

**FIGURE 3:**
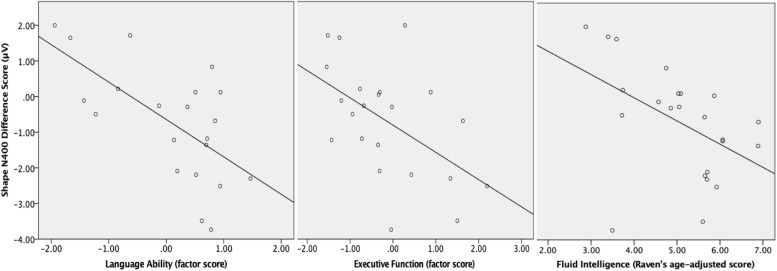
Scatter plots of relation between the shape N400 difference score (negative difference score represents a larger N400 incongruity effect) and the three outcome measures at 6–7 years.

Instead of the original 15 outcome variables, our main analyses focused on three cognitive variables at age 6–7 years: the latent variables “language ability,” “and “verbal executive function” as well as the observable variable “fluid intelligence” (see section “Materials and Methods”). These three cognitive variables correlated with each other: language ability and fluid intelligence were most strongly related *(r* = 0.681, *p* < 0.001), followed by verbal executive function and fluid intelligence *(r* = 0.42, *p* = 0.012), and language ability and verbal executive function (*r* = 0.487, *p* = 0.003). Table 4 reports all bivariate longitudinal correlations with the three outcome factors, and scatter plots between the shape N400 effect at 20 months and three outcome factors can be seen in Figure 3.

**TABLE 4:** Longitudinal correlations between toddler measures and latent factors in school age.

	**Language ability**	**Verbal Executive Function**	**Fluid intelligence**
			
	**PEARSON**	**PEARSON**	**PEARSON**
Shape N400 20 m	**−0.595^∗∗^**	**−0.526^∗^**	**−0.477^∗^**
*p-value*	*0*.*003*	*0*.*012*	*0*.*021*
*n*	*23*	*23*	*23*
Shape late neg. 20 m	0.307	**−0.751^∗∗∗^**	–0.086
*p-value*	*0*.*165*	> 0.001	*0*.*696*
*n*	*23*	*23*	*23*
Productive vocab. 24 m	**0.508^∗∗^**	0.148	**0.393^∗^**
*p-value*	*0*.*005*	*0*.*435*	*0*.*029*
*n*	*29*	*30*	*31*
Receptive vocab. 20 m	**0.419^∗^**	0.200	**0.451^∗^**
*p-value*	0.019	0.273	0.008
*n*	31	32	33

*Bold font indicates correlations that are statistically significant (*p* < 0.05). ^∗^*p* < 0.05, ^∗∗^*p* < 0.03, and ^∗∗∗^*p* < 0.01.*

With a regular alpha-level of 0.05, the shape N400 component strongly predicted language ability, but also verbal executive function and fluid intelligence. The relation between the shape N400 and language ability remained significant after controlling for verbal executive function (*r* = −0.500, *p* = 0.025) and fluid intelligence (*r* = −0.454, *p* = 0.044). The late negative ERP component in the shape condition strongly predicted verbal executive function, but not language ability or fluid intelligence. Two of the behavioral measures of early vocabulary size, receptive vocabulary at 20 months and productive vocabulary at 24 months also correlated significantly with later language ability, as well as fluid intelligence, while productive vocabulary at 20 months was unrelated to any of the outcome factors. Since each toddler variable was tested for correlations with three outcome variables, a more cautious approach would be to adjust for multiple comparisons by setting the alpha-level to 0.05/3 = 0.017. After such adjustment, the main significant correlations were as follows: the shape N400 component significantly predicted language ability and verbal executive function; the late negative ERP component in the shape condition predicted verbal executive function; productive vocabulary at 24 months predicted language ability, while receptive vocabulary at 20 months predicted fluid intelligence.

A linear regression model demonstrated that the two predictors Shape N400 effect at 20 months and productive vocabulary size at 24 months together explained 57% (adjusted *R*^2^ = 0.519) of the variance in Language Ability at 6–7 years [*F*(2,16) = 10.72, *p* = 0.001)]. Both predictors made unique contributions to the model, shape N400 explaining 40% of the variance and productive vocabulary 15% shape N400: β = −0.633, *t*(2,19) = −3.87, *p* = 0.001; productive vocabulary: β = 0.384, *t*(2,19) = 2.349, *p* = 0.032).

## Discussion

This study investigated individual differences in electrophysiological measures of semantic processing and object recognition in toddlers and how they relate to the children’s cognitive development 4–5 years later. The results show that the regular N400-effect, which in our paradigm depends on both semantic processing of words and recognition of common objects, did not differentiate between children on any future measures of language or cognition. The incongruity responses related to object shape recognition, however, correlated with several measures at 6–7 years. These incongruity responses provide an electrophysiological measure of the toddlers’ ability to recognize common objects from only overall shape. Thus, relations between these toddler and school-age measures show that individual differences toddlers’ shape recognition can predict future cognitive skills. To understand the pattern of correlations, and to improve the psychometric properties of our measures by creating normally distributed variables, we used a principal components analysis, where the cognitive tests clustered into two factors. We will argue that it is reasonable to understand one factor as *language ability* and the other factor as *verbal executive function*. In addition to these two constructs, we had *fluid intelligence* as a separate outcome variable (measured by the Raven’s colored progressive matrices test). We demonstrated that our three outcome factors at 6–7 years were predicted by different temporal segments of the ERP response elicited by detection of incongruity between object shape and a verbal label. Individual differences in the mid-latency N400 effect predicted all three outcome factors, while a later, more centrally distributed effect strongly predicted verbal executive function specifically.

### Understanding the Outcome Variables

The tests that most clearly differentiated between the two factors were TROG-2 (receptive grammar comprehension) and expressive vocabulary on the one hand (factor 1), and digit span backward on the other hand (factor 2). The other tests that were included in factor 1 were similarities expressive (abstract conceptual reasoning) and non-word repetition. Thus, factor 1 clearly involves both receptive and expressive language skills, and both semantic, syntactic and phonological processing. Therefore, it seemed reasonable to interpret the factor as general *language ability.* Since factor 2 loaded most heavily on the digit span backward task, and also on the forward digit span task, it is strongly related to processes involving short-term and working memory, both maintenance and manipulation processes. However, the third test clustered in factor 2 was speeded naming, specifically the accuracy measure (not time) of the test. In speeded naming, children are required to name different shapes, colors and both colors and shapes (e.g., “green square”) as quickly as possible. Although it is described in the NEPSY manual as a language test of rapid semantic access, it is a complex task that requires both shifting of attention from one stimulus to the next, and suppression or inhibition of the previously activated word to replace with the activation of the next word. Since factor 2 includes tests that involve both working memory and other processes of cognitive control, we chose to label it as *verbal executive function.* Executive functions encompass a broad range of cognitive functions, and we do not claim to be able to specify that factor 2 captures a particular aspect of these. There is reasonable agreement that executive functions can be divided into three main domains: inhibition, working memory, and cognitive flexibility (Diamond, 2013), and the tests included in factor 2 are considered to rely heavily on at least working memory and inhibition, and involve verbal tasks that are relevant to language processing. Unfortunately, the test battery did not include specific tests of cognitive flexibility, so it is difficult to say if such a test also would cluster clearly into factor 2.

Finally, the third outcome measure used in the analyses was Raven’s Colored Progressive Matrices. We chose to include this test in the study because the Raven’s matrices series of tests is the most widely used measure of fluid intelligence. The concept of fluid intelligence refers to the ability to solve novel problems using logical reasoning, and the matrix task is a non-verbal measure of this ability (Horn and Cattell, 1966). Since this test was included in the study in order to capture an ability distinct from language and working memory processes, it was not included in the PCA but rather remained as a separate variable.

### Shape Recognition

A main conclusion from our results is that brain measures of shape recognition in toddlers predict language ability 4–5 years later. Toddlers with larger N400 incongruity effects in the shape conditions developed better language skills. However, the regular N400 incongruity effect was unrelated to language development. This pattern of results indicates that the process of quickly recognizing object silhouettes and mobilizing the associated verbal label is more predictive of future language development than general efficiency of semantic processing. Importantly, the incongruity response during the shape condition depends on more than simple vocabulary knowledge (captured by the regular object condition), rather it requires that the child has paid attention to the global shape properties of the object. This finding extends previous research on the children’s shape recognition, which has established that toddler’ language development is associated with an improvement in shape recognition and the subsequent development of a shape bias (Smith, 2003; Pereira and Smith, 2009; Yee et al., 2012). The ability to extract the global shape of objects from visual input is thought to enable early word learning by directing attention to a relevant dimension for object categorization. Why then, would early individual differences in this ability be relevant for language skills in school-aged children? One possible explanation is that early abilities provide important prerequisites for future development. If toddlers who are early to attend to the most relevant visual properties of objects and link these rapidly with verbal labels, are more efficient word learners early on, their larger vocabularies may have a snowball effect on their subsequent language development. It is also possible that a specific task at a certain age, like for example shape recognition around 20 months, depends heavily on a cognitive ability, such as visual categorization/abstraction, that is also important in other tasks at other ages. Individual differences in that cognitive ability may be relatively stable and therefore predict variation in language and verbal executive function skills later in life.

Our analysis of the ERP data indicates that the task of recognizing objects and quickly processing whether an object is semantically related to a word involves at least two distinct stages of processing that are affected by the semantic relatedness. The first stage, captured by a classic N400 effect, may be reasonably interpreted as a stage of lexical access, where the picture of an object primes the associated word and thus facilitates lexical access of words in congruous presentations (for reviews on the N400, see Lau et al., 2008; Kutas and Federmeier, 2011). The fact that only lexical priming by object shape was predictive of future language skills highlights the importance of skills related to categorization and generalization in the early stages of language development. In the regular object conditions, children were presented with the same picture that had been presented five times previously and paired with the same word. Efficiency of lexical access during these conditions was not predictive of language development. However, in the shape conditions the children were required to quickly interpret a completely novel stimulus, which was much more abstract than previous stimuli, and in addition activate the associated word.

There also seems to be a second stage of processing the semantic information, indexed by a late ERP component also demonstrating an incongruity effect. Although it is not clear whether this effect can be classified as a particular established ERP component, later stage ERP components are often interpreted as involving contextual integration or memory updating (Reynolds and Romano, 2016). It is especially interesting that this later component so strongly predicted our second factor of cognitive abilities conceptualized as verbal executive function. The verbal executive function factor loaded particularly strong on the digit span backward test of working memory. The strong correlation between the late ERP component and the verbal executive function factor suggests that the late component could reflect a stage of verbal working memory, perhaps where the presented word is compared to an internally activated lexical item primed by the picture. Children with better working memory skills may be more actively maintaining a pre-activated lexical item and comparing it to the actual presented word (see Vales and Smith, 2015). The importance of working memory even in early word processing has been suggested previously by results showing that toddlers’ speed of word recognition in a looking-while-listening task predicts working memory in school-aged children (Marchman and Fernald, 2008).

To our knowledge there are no previous reports that N400 responses have predictive value over such a long period of time. Other ERP responses related to word processing have been shown to capture individual differences in language ability, although few studies show longer-term predictive value. The N200-500 component, which relates to word form familiarity or word recognition (for a review, see Friedrich, 2011) has been related to linguistic maturity in both normally and atypically developing children. For instance, 10-month-olds’ N200-500 response to words predicts vocabulary size at 12 months and 24 months, as well as performance on a “looking-while-listening” word comprehension test at 16 months (Junge et al., 2012b; Junge and Cutler, 2014). When measured in 2-year-olds with autism, the word recognition response has been shown to predict receptive language skills, cognitive ability, and adaptive behavior up to 6 years (Kuhl et al., 2013). Our results demonstrate for the first time that electrophysiological measures of semantic processing (the N400) in young typically developing children can be predictive of individual differences in language ability many years later.

We know from previous research that the stability of rank order in language ability in young children is fairly low, which makes it difficult to use toddler vocabulary measures to predict language delays even a couple of years later (Feldman et al., 2005; Westerlund et al., 2006; Henrichs et al., 2011). Some of this instability is likely due to issues of reliability of the behavioral measures used with young children. Since children’s results on behavioral tests depend their cooperative abilities, “online” measures, which measure processing in real time rather than children’s end-point responses, have important potential to capture individual differences in cognition. Neural measures such as ERP responses have such potential, as well as for example eye movement measures such as in the “looking-while-listening”-paradigm (e.g., Marchman and Fernald, 2008). Although the predictive value of ERP responses as demonstrated in this study is still highly tentative and requires much further research to be established, these measures may be able to capture individual differences in processing that are difficult to measure otherwise.

### Methodological Considerations

The cognitive test battery used in the present study merits some consideration. The PCA analysis of the selected outcome measures resulted in two latent factors labeled language ability and verbal executive function. The factor language ability loaded most heavily on the three following variables: language comprehension (comprehension of grammar), expressive vocabulary and non-word repetition. This did not come as a surprise since these tests are highly important predictors of language development. Non-word repetition not only predicts vocabulary development, but is even considered a clinical marker of developmental language disorder in children this age (Bishop et al., 2017). Together with language comprehension (assessed with the TROG-2), non-word repetition, and lexical organization are considered clinical markers for language disorder in Swedish children already at age four (Lavesson, 2012). In Lavensson’s study, these variables turned out to have higher sensitivity and specificity than a range of other measures tapping several different language areas.

As is often the case with longitudinal studies, the present study has weaknesses related to data attrition and the risk of obtaining false positive results due to many data variables. The most important results reported rest on a data-set of 23 participants. A sample of this size renders the correlational analyses low-powered and increases the risk of false positive results. On the other hand, the type of data we offer, electrophysiological developmental data as well as longitudinal data from toddlerhood to school entry, is rare and therefore may illuminate relationships which have not been observed previously. The choice of using principal components analyses for both the ERP data and the cognitive data was an attempt to improve properties of the data sets. For the ERP data, the results of the PCA are potentially less noisy and more precise than alternative measures such as mean difference amplitude across a certain time window. For the test measures at 6–7 years, the PCA resulted both in fewer variables (which decreases the risk of false positive correlations) and variables with better statistical properties (normally distributed) so that better methods for data analysis could be used. The many bivariate correlations calculated in Table 3 are reported in order to show the full pattern of longitudinal relationships. Of course, individual correlations must be interpreted carefully considering that the alpha-level has not been corrected for multiple comparisons. However, the main findings that we emphasize are the results of the regression analyses between larger data components, and are worth to be taken seriously. Still, the study is explorative and the relationships we suggest need to be confirmed by future research.

## Conclusion

The current study provides the first evidence that electrophysiological responses in tasks measuring semantic processing have predictive value from toddlerhood to early school age. A measure of object shape recognition at age 20 months was found to predict language abilities and verbal executive functions 4–5 years later, even when language abilities in toddlerhood were taken into consideration. Electrophysiological measures of general object recognition were not associated with later language and cognitive abilities, suggesting that the predictive value was specific to object shape.

## Data Availability

The datasets generated for this study are available on request to the corresponding author.

## Ethics Statement

All subjects gave written informed consent in accordance with the Declaration of Helsinki. The protocol was approved by the Institutional Review Board at the Section of Logopedics, Phoniatrics, and Audiology, Lund University.

## Author Contributions

KB had overall responsibility for the study design, collecting all the data at 20 months, as well as all data processing, statistical analysis, and writing of the first draft of the manuscript. BS led the designing of the follow-up study and supervising the students who performed the standardized tests at 6–7 years. JT and ML were highly involved in the designing of the ERP study, and analyzing and interpreting of the ERP data. All authors contributed to interpreting all the results, and reviewing and editing of the manuscript.

## Conflict of Interest Statement

The authors declare that the research was conducted in the absence of any commercial or financial relationships that could be construed as a potential conflict of interest.

## Footnotes

1 The toddler data presented in this paper were part of a large project investigating several different aspects of semantic processing and word learning at 20 and 24 months, and the results have been reported previously (Borgström et al., 2015a, b, 2016). The present study focused on the relation between these toddler data and behavioral data from the same children that were collected 4½–5 years later. The analyses of toddler data were conducted on a subset of the original data which concerned shape recognition specifically, and we performed novel analyses which were better suited to the longitudinal research questions. The section “Materials and Methods” still reports the entire procedure so that readers are able to understand the experimental context.

2 www.clipart.com
