# Supplementary material for: Brain Measures of Toddlers’ Shape Recognition Predict Language and Cognitive Skills at 6–7 Years
Source: Front Psychol. 2019 Aug 23;10:1945. doi: 10.3389/fpsyg.2019.01945 (PMC6716541; doi:10.3389/fpsyg.2019.01945)
Supplement: Supplementary file 1 [file Table_1.docx]

Supplementary Material 1

*Longitudinal correlations between original ERP measures (in Borgström et al., 2015a) and outcome measures at 6-7 years*

|  | TROG | Expressive Vocab. | Similarities Expressive | Language Ability | Verbal Executive Function | Fluid Intelligence |
| --- | --- | --- | --- | --- | --- | --- |
|  | SPEARMAN | SPEARMAN | SPEARMAN | PEARSON | PEARSON | PEARSON |
| **Shape N400 Parietal (500-900 ms)** | **-.442*** | **-.450*** | **-.606**** | **-.687***** | -**.492**** | **-.597**** |
| *p-value* | *.035* | *.031* | *.002* | *< .001* | *.020* | *.003* |
| *n* | *23* | *23* | *23* | *23* | *23* | *23* |

|  | Digit Span Forward | Digit Span Backward | Similarities Receptive | Speeded Naming | Auditory Analysis | CCC2 |
| --- | --- | --- | --- | --- | --- | --- |
|  | SPEARMAN | SPEARMAN | SPEARMAN | SPEARMAN | SPEARMAN | SPEARMAN |
| **Shape N400 Parietal (500-900 ms)** | -.357 | -.350 | -.301 | -.331 | -.139 | -.253 |
| *p-value* | *.095* | *.101* | *.162* | *.123* | *.526* | *.244* |
| *n* | *23* | *23* | *23* | *23* | *23* | *23* |

|  | Digit Span Forward | Digit Span Back | Language Ability | Verbal Executive Function | Fluid Intelligence |  |
| --- | --- | --- | --- | --- | --- | --- |
|  | SPEARMAN | SPEARMAN | PEARSON | PEARSON | PEARSON |  |
| **Shape N400 Central (500-900 ms)** | **-.541**** | **-.668***** | **-.453*** | **-.671***** | -.356 |  |
| *p-value* | *.008* | *<.001* | *.039* | *<.001* | *.096* |  |
| *n* | *23* | *23* | *23* | *23* | *23* |  |

|  | TROG | Expressive Vocabulary | Similarities Receptive | Similarities Expressive | Speeded Naming | Auditory Analysis | CCC2 |
| --- | --- | --- | --- | --- | --- | --- | --- |
|  | SPEARMAN | SPEARMAN | SPEARMAN | SPEARMAN | SPEARMAN | SPEARMAN | SPEARMAN |
| **Shape N400 Central (500-900 ms)** | -.270 | -.064 | -.257 | -.402 | -.181 | -.169 | -.237 |
| *p-value* | *.213* | *.770* | *.237* | *.057* | *.408* | *.441* | *.276* |
| *n* | *23* | *23* | *23* | *23* | *23* | *23* | *23* |

Note. The shape N400 central effect between 700-900 ms that correlated with concurrent vocabulary at 20 months, also did not correlate with Expressive Vocabulary at 6-7 years ( *r* = -.064, *p* = .770).
