# Supplementary material for: Brain Measures of Toddlers’ Shape Recognition Predict Language and Cognitive Skills at 6–7 Years
Source: Front Psychol. 2019 Aug 23;10:1945. doi: 10.3389/fpsyg.2019.01945 (PMC6716541; doi:10.3389/fpsyg.2019.01945)
Supplement: Supplementary file 2 [file Table_2.DOCX]

Supplementary material 2.

*Longitudinal Correlations Between Toddler and School-age Measures*
